# Indoor terpene emissions from cooking with herbs and pepper and their secondary organic aerosol production potential

**DOI:** 10.1038/srep36623

**Published:** 2016-11-10

**Authors:** Felix Klein, Naomi J. Farren, Carlo Bozzetti, Kaspar R. Daellenbach, Dogushan Kilic, Nivedita K. Kumar, Simone M. Pieber, Jay G. Slowik, Rosemary N. Tuthill, Jacqueline F. Hamilton, Urs Baltensperger, André S. H. Prévôt, Imad El Haddad

**Affiliations:** 1 Laboratory of Atmospheric Chemistry, Paul Scherrer Institute, Villigen, 5232, Switzerland; 2 Wolfson Atmospheric Chemistry Laboratories, University of York, York, YO10 5DD, UK

## Abstract

Cooking is widely recognized as an important source of indoor and outdoor particle and volatile organic compound emissions with potential deleterious effects on human health. Nevertheless, cooking emissions remain poorly characterized. Here the effect of herbs and pepper on cooking emissions was investigated for the first time to the best of our knowledge using state of the art mass spectrometric analysis of particle and gas-phase composition. Further, the secondary organic aerosol production potential of the gas-phase emissions was determined by smog chamber aging experiments. The emissions of frying meat with herbs and pepper include large amounts of mono-, sesqui- and diterpenes as well as various terpenoids and p-cymene. The average total terpene emission rate from the use of herbs and pepper during cooking is estimated to be 46 ± 5 *gg*^-1^_Herbs_ *min*^-1^. These compounds are highly reactive in the atmosphere and lead to significant amounts of secondary organic aerosol upon aging. In summary we demonstrate that cooking with condiments can constitute an important yet overlooked source of terpenes in indoor air.

More than three million people die prematurely each year from outdoor air pollution, more than malaria and HIV combined, and without action the number of deaths will double by 2050^1^. In response to this, substantial scientific effort has been devoted to the real-time determination of the chemical composition and the sources of the urban organic aerosols, which are believed to be a major cause of these premature deaths. Comparatively, the characterization of indoor air pollution has received less attention, although we spend up to ninety percent of our time indoors, where the air can be orders of magnitudes more polluted. The main sources of primary particles indoors in developing countries are biomass burning emissions^2^ while in developed countries cooking emissions are thought to be the main contributor^3^. In addition to high indoor levels of directly emitted primary organic aerosols, deleterious non methane organic gases (NMOG, e.g. formaldehyde) such as monoterpenes (e.g. limonene) are also present. These compounds are thought to originate primarily from detergent use^4^,^5^,^6^,^7^ and are now believed to be the main source of indoor secondary organic aerosols, upon reaction^8^,^9^. Both secondary organic aerosol (SOA) and aged NMOGs from terpenes may have deleterious effects on human health^7^,^10^, demonstrating the importance of identifying indoor terpene sources. Cooking processes have been recognized as major contributors to particulate matter (PM) concentrations in indoor^11^,^12^,^13^,^14^ and outdoor^15^,^16^,^17^,^18^,^19^ environments. In addition to PM, these processes have been shown to emit large amounts of NMOGs^20^,^21^,^22^,^23^. Most NMOG reported in previous studies are aldehydes from frying process, however the impact of NMOG emissions from other cooking processes on indoor gas and particle phase concentrations has never been assessed. Using two-dimensional gas chromatography time-of-flight mass spectrometry (GC × GC-ToF-MS), the first measurements of the volatile and intermediate volatility compounds released upon heating pepper and common herbs were made. Real-time characterization of aerosol and gas phase composition of frying meat with different amounts of herbs and/or pepper was performed using state of the art on-line mass spectrometeric techniques, including a proton transfer reaction time-of-flight mass spectrometer (PTR-ToF-MS) and a high-resolution time-of-flight aerosol mass spectrometer (HR-ToF-AMS). In this study we (i) identify and quantify terpene emissions from cooking processes, (ii) estimate their SOA production potential and (iii) evaluate their impact on indoor air quality.

## Results and Discussion

### Identification and quantification of gaseous emissions from condiment use

**Figure 1:**
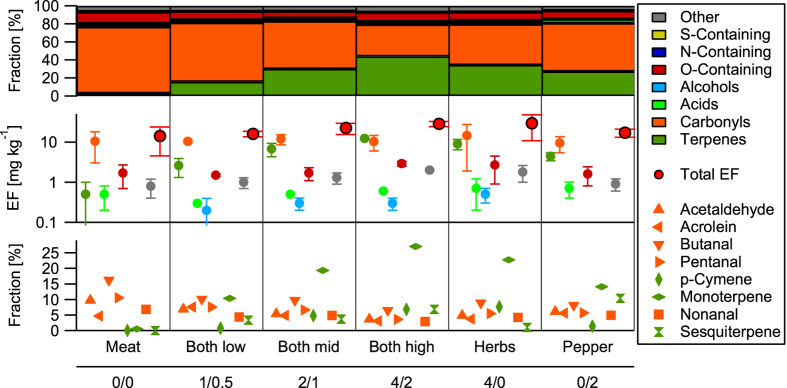
Relative composition (upper panel), emission factors (middle panel) and compounds contributing more than 5% to the total mass (lower panel) for pan frying of beef with canola oil and varying amounts of seasoning as measured with the PTR-ToF-MS. The upper axis label indicates the kind of experiment (Table S3) and the lower axis label indicates the amount of seasoning added in grams (herbs/pepper). Every experiment was repeated once. Error bars represent the range of the results.

**Figure 2:**
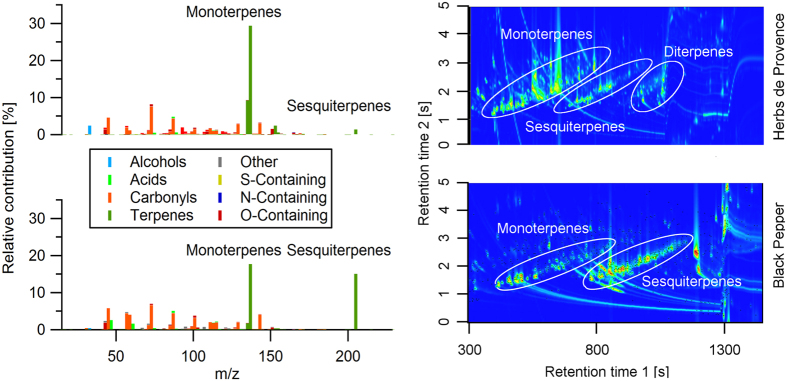
Comparison of PTR-ToF-MS mass spectra (left side) and GC × GC-ToF-MS chromatograms (right side). Upper panels are measurements of “Herbs de Provence” and lower panels of black pepper. GC × GC-ToF-MS chromatograms derive from volatilizing the seasoning at 180 °C. PTR-ToF-MS mass spectra derive from frying the seasoning together with meat at 180 °C.

**Figure 3:**
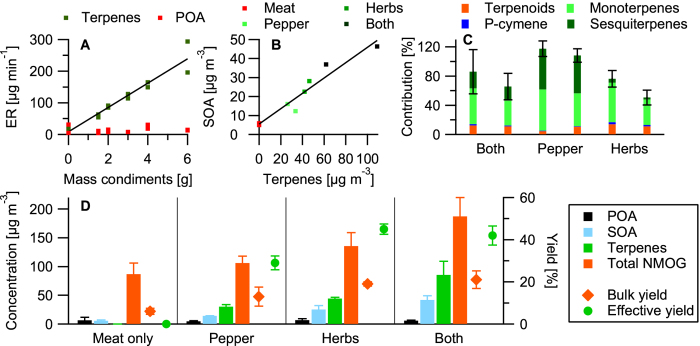
(**A**) Terpene emissions per gram seasoning (Table S3), (**B**) Secondary organic aerosol (SOA) formed from different amounts of terpenes in the chamber and (**C**) contribution of different terpene species to SOA (double columns represent experiment repeats). (**D**) Gives an overview of primary organic aerosol (POA), SOA, and terpene concentrations in the chamber for all experiment conditions and calculated bulk and effective yields (mass SOA formed divided by mass terpenes reacted). Every experiment was repeated once. Error bars in (**C**) represent the measurement error and the uncertainties resulting from the literature yields. Error bars in (**D**) represent the range of results.

Figure 1 shows the chemical composition and emission factors (EF) for pan-frying lean beef in canola oil with varying amounts of grained black pepper and “Herbs de Provence” (from now on called “herbs” including rosemary 20%, savory 26%, oregano 26%, thyme 19% and basil 3%). Compounds measured with the PTR-ToF-MS were categorized as described in Klein *et al*.^23^ with a new class “terpenes” including p-cymene (C_10_H_14_), monoterpenes (C_10_H_16_), terpenoids (C_10_H_14_O, C_10_H_16_O) and sesquiterpenes (C_15_H_24_). The emission of S- and N-containing compounds were below 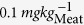 for all experiments. Release of acids and alcohols was negligible with EFs below 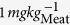. The emissions of carbonyls were comparable for all experiments with an EF of 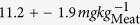, indicating that herbs emit negligible amounts of carbonyls upon heating compared to meat frying. The observed carbonyls are mainly aldehydes and are comprised of large amounts of hexanal (C_6_H_12_O) and nonanal (C_9_H_18_O) from the decomposition of heated oils and butanal (C_4_H_8_O) emitted from the meat^23^. The addition of 2, 4 and 6 g of condiments to the meat before frying for 10 minutes led to terpene emission of 2.6, 6.8 and 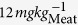 respectively. A table with all emission factors can be found in the supplementary information (Table S4). Figure 2 shows a comparison of the GC × GC-ToF-MS chromatograms and the PTR-ToF-MS spectra of pepper and herbs. The good agreement between both techniques enables us to identify the dominant contributors to the bulk signal measured online by PTR-ToF-MS. Both measurement techniques show that the herb mixture mostly emits p-cymene and monoterpenes but only low amounts of sesquiterpenes. Significant amounts of oxygenated sesquiterpenes such as caryophyllene oxide (C_15_H_24_O) or cadinol (C_15_H_26_O) and diterpenes e.g. cembrene (C_20_H_32_) were detected in the herbs emissions by GC × GC-ToF-MS but not by PTR-ToF-MS. This results in different C_10_/C_15_ compound ratios of 20 for the PTR-ToF-MS measurements and 2.5 for the GC × GC-ToF-MS measurements. The monoterpenes from herbs are dominated by limonene, camphene and γ-terpinene and the sesquiterpenes are dominated by *β*-caryophyllene, bisabolene and cadinene as observed from GC × GC-ToF-MS intensities. Black pepper emits mostly sesquiterpenes, lower amounts of monoterpenes, and almost no p-cymene. For black pepper the C_10_/C_15_ compound ratios from PTR-ToF-MS and GC × GC-ToF-MS measurements agree (1.4 and 1.25, respectively). The dominant monoterpenes emitted from grained and heated black pepper are α-pinene, ocimene and limonene, and the dominant sesquiterpenes are *β*-caryophyllene and copaene. A complete list of all the terpenes identified by GC × GC-ToF-MS can be found in the supplementary information (Tables S1 and S2). Although significant differences in herb and pepper emissions exist, both types of condiments emit similar amounts of terpenes (excluding diterpenes and oxygenated sesquiterpenes), with an average emission rate of 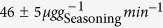 (Fig. 3A). Primary NMOG emissions observed in the smog chamber compared well with direct emissions from the same experiments, indicating a good sample transmission into the chamber (Fig. S1). The increasing amount of total primary NMOG in the chamber (Fig. 3D) is a direct result of the increased amount of seasoning added to the meat; a linear increase (*R*^2^ = 0.95) of terpene emissions from 67 to 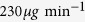 was observed with the amount of seasoning added ranging from 1.5 to 6 g (Fig. 3A).

### SOA formation potential of herb emissions

The HR-ToF-AMS mass spectra of primary organic aerosol (POA) emitted from frying meat with oil alone resembles that of frying meat with herbs and pepper (Fig. S2). POA concentrations in the chamber were 6.0 ± 3.3 *μgm*^−3^ for all experiments (Fig. 3D), independent of the amount of seasoning added, showing that POA predominantly resulted from meat frying. During aging, almost all terpenes and terpenoids reacted to form low-molecular weight acids and carbonyls (Fig. S3) and SOA. The average terpene half-life in the chamber was around 15 min, consistent with the extremely rapid SOA production. While these half-life values are fairly reproducible between the different experiments (~25% variability), the estimated reaction rates of the identified terpenes against both OH and O_3_ (*k*_OH_ and 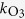, respectively) span almost 2 orders of magnitude. In order to determine the average *k*_OH_ and 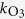 for the terpene mixture in the chamber, we have used a Monte Carlo simulation to generate a probability density function of the *k*_OH_-to-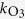 ratio, assuming a randomly generated mixture of the most important mono- and sesquiterpenes. Using this ratio, the calculated terpene half-life distributions, and the OH and O_3_ concentrations (2.5 × 10^7^ and 1 × 10^12^ *molecules* *cm*^−3^, respectively), we estimated a k_OH_ and 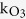 of 2.9 × 10^−11^ *molecules*^−1^ *cm*^3^ *s*^−1^ (32% error) and 2.5 × 10^−16^ *molecules*^−1^ *cm*^3^ *s*^−1^ (54% error), respectively. We have found that under our chamber conditions 73 ± 8% of the terpenes will react with OH, whilst in indoor and outdoor air, the reaction with O_3_ will dominate. The total amount of SOA (SOA_tot_) formed ranged from 4.9 to 64.4 *μgm*^−3^ and strongly depended (*R*^2^ = 0.93, slope = 0.4) on the initial mass of terpenes in the chamber (Fig. 3B). To calculate the terpene SOA effective yields and their contribution, the SOA formed due to the addition of seasoning needs to be calculated (SOA_cond_). This is achieved by subtracting the average SOA formed by the emissions from frying meat with oil alone from SOA_tot_ for every experiment. The average effective SOA yield (mass SOA_cond_ divided by mass terpenes reacted) from the terpenes was 40%. Interestingly the effective yield from herbs terpenes of 45% is higher than the effective yield from pepper terpenes of 29% (Fig. 3D). A possible explanation is that herb emissions contain more diterpenes and oxygenated sesquiterpenes as indicated by GC × GC-ToF-MS measurements (Fig. 1), not detected with the PTR-ToF-MS. These terpene classes are likely efficient intermediate volatility SOA precursors and would contribute to the effective SOA yields calculated from the herbs terpenes. For all experiments the terpenes measured with the PTR-ToF-MS can account for between 50 to 120% of SOA_tot_ (Fig. 3C). The contributions of the individual terpene families were estimated using literature yields^24^,^25^. The unexplained SOA_tot_ can originate from the measurement and literature yield uncertainties, other SOA producing compounds and the diterpenes and oxygenated sesquiterpenes not detected with the PTR-ToF-MS (especially for the herbs experiments). Monoterpenes dominated the SOA formation potential from herb emissions (35–54%) with a substantial contribution from the terpenoids (11–14%). For pepper emissions, mono- and sesquiterpenes contributed equally to SOA formation (45–56% vs. 52–56%). P-cymene contributed less than 2% to the observed SOA for all experiments.

### Environmental impact

Previous studies showed that gaseous aldehyde emission factors of frying processes are sufficiently high to generate concentrations that exceed legal or workplace limits in kitchens without ventilation^23^. However, no estimate of the influence of terpene emissions from cooking processes on indoor air quality currently exists. Because of short cooking times, air exchange will only have a minor effect (<20%) on peak concentrations and therefore will not be taken into account. The average volume of a kitchen in Europe is about 25 *m*^3^, and the average number of persons per household in the EU-28 is 2.3^26^. The per capita use of seasonings in the EU is about 900 g per year^27^, and the average terpene emissions from frying meat with seasoning for 10 minutes are 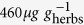. Assuming that half of the daily amount of herbs are used to cook one meal and neglecting air exchange or oxidation during the 10 minutes cooking time, the total terpene concentration in the kitchen of an average European household is approximately 50 *μgm*^−3^. Muniz *et al*.^28^, report the mean and median concentration of *α*-pinene from 21 studies in indoor and school environments to be 32.8 and 11 *μgm*^−3^, respectively. The mean and median concentration of limonene from 24 studies is reported to be 20.6 and 19.2 *μgm*^−3^, respectively. This indicates that terpene concentrations measured in indoor environments, which are currently mostly attributed to cleaning detergents^7^, could be strongly influenced by cooking with herbs. This implies that measuring only *α*-pinene and limonene is not sufficient for estimating the total terpene loads indoors, suggesting other potential terpene emissions should also be considered (mono-, sesqui-, diterpenes and terpenoids). Our calculations show that terpene emissions from cooking with seasoning are of comparable magnitude to those from detergent use. So far indoor air quality studies have only estimated SOA from monoterpenes (mostly limonene)^8^,^9^. If the highly reactive terpene mixture from cooking with seasonings is included, the modeled SOA formation indoors could increase even though the majority of the precursor emissions will be lost by air exchange. By considering air exchange rates (around 0.7 h^−1^) and oxidant concentrations typical of indoor environments O_3_=4 ppb and OH = l*e*^5^ *molecules*^−1^ *cm*^3^ *s*^−1^ 9 we estimate that only 5% of the terpenes would react to form SOA, while the majority will be lost by dilution. In summary our results indicate that cooking with seasoning is an important yet overlooked source of terpenes and SOA indoors and should be considered for future indoor air quality studies.

## Methods

### Experimental set up

The pan frying was conducted on an electrical heating plate, which was situated in a 0.06 *m*^3^ metal housing. The emissions were pumped from the housing at a rate of 14 Lmin^−1^, approximately 2 Lmin^−1^ of this was diluted through a heated (200 °C) ejector diluter (DI-1000, Dekati Ltd., Kangasala, Finland). In the ejector diluter, the emissions were diluted with pure air (737-250 series, Aadco Instruments Inc., U.S.A.) by about a factor 1:10. For the direct measurements, the emissions were subsequently diluted before analysis by another factor of 1:10 with a second unheated ejector diluter. For the aging experiments, the emissions that had been diluted once were introduced into a 7 *m*^3^ Teflon smog chamber (described in Bruns *et al*.^29^) situated in a temperature controlled housing regulated to 20 °*C*. The NMOG, methane (CH_4_) and the particulate matter (PM) were measured with a highly sensitive proton transfer reaction time-of-flight mass spectrometer (PTR-TOF-8000, Ionicon Analytik Ges.m.b.H., Innsbruck, Austria), a CO_2_/CO/CH_4_/H_2_O analyzer (G2401, Picarro Inc., Santa Clara, U.S.A.) and a high resolution time-of-flight aerosol mass spectrometer (HR-Tof-AMS, Aerodyne Research Inc., Billerica, U.S.A.), respectively.

### Experimental procedure

For each experiment, two pieces of lean beef (~250 g) were seasoned with varying amounts of “Herbs de Provence” (McCormick, Promena AG; including rosemary 20%, savory 26%, oregano 26%, thyme 19% and basil 3%) and/or grained black pepper (Qualité & Prix, Coop AG). The pan was heated to 180 °C with 5 g of canola oil and after reaching this temperature the meat was fried on both sides for 5 minutes. The direct measurements were conducted continuously while the injection into the smog chamber was only whilst the meat was in the pan. After the emissions were well equilibrated in the smogchamber, 4 sets of 10 UV lights (90–100 W, Cleo Performance, Philips) situated around the smogchamber, were switched on to induce aging. To further increase the amount of aging, nitrous acid (HONO) in N_2_, which forms OH radicals via photolysis, was introduced into the chamber at a flow rate of about 2 Lmin^−1^. The HONO was produced as described in Taira *et al*.^30^ from H_2_SO_4_ and NaNO_2_ in a glass reactor. In order to monitor the amount of aging in the smog chamber, 2 *μL* of d9-butanol (butanol-D9, 98%, Cambridge Isotope Laboratories) were introduced into the chamber through a heated line. By measuring the decay of the d9-butanol, using 3.4 × 10^−12^ *cm*^3^ *molec*^−1^ *s*^−1^ as the rate constant with respect to OH^31^, the OH concentration in the chamber can be calculated. After each aging experiment the smogchamber was cleaned by filling it with O_3_ and humid air, then turning on the UV lights for at least 1 hour. Afterwards the smogchamber was flushed with pure dry air over night. Before the next injection, the chamber was partially filled with pure humid air again. All aging experiments were conducted at 50% relative humidity and a temperature of 20 °C. A Gerstel Twister autosampler and a Gerstal thermal desorption unit (TDU) were used to analyse herbs and pepper by GC × GC-ToF-MS. Either 10 mg of herbs or 5 mg of pepper were packed inside thermal desorption tubes between 2 pieces of glass wool. The samples were heated from 80 °C to 180 °C at 60 °C/*min*, and then held isothermally for 5 minutes before being transferred from the TDU to the cooled injection system (CIS). The TDU transfer line temperature was 200 °C. The CIS was heated from −80 °C to 280 °C at 10 °C per minute before the sample was introduced into the GC × GC system.

### Instrumentation

The PTR-ToF-MS accesses NMOG with a proton affinity higher than that of water^32^. The NMOG are protonated by the use of H_3_O^+^ ions and subsequently measured using a time-of-flight mass spectrometer (Tofwerk AG, Thun, Switzerland). A detailed description of the instrument can be found in Jordan *et al*.^33^. Operating conditions as well as calibration procedures of the PTR-ToF-MS during the measurement campaign are described in Klein *et al*.^23^. The HR-Tof-AMS provides real-time (<1 min) quantification of the size-resolved mass and chemical composition of non-refractory sub-micron aerosol particles with a vacuum aerodynamic diameter below 1 *μm*^34^. By using a PM^2.5^ lens^35^ we were able to measure particles with a vacuum aerodynamic diameter up to 2.5 *μm*. A detailed description of the instrument and data treatment procedures can be found in DeCarlo *et al*.^36^. The GC × GC-Tof-MS measurements were conducted using a Gerstel thermal desorption unit (Mülheim an der Ruhr, Germany) coupled to a GC × GC-TOF-MS system, incorporating an Agilent 6890 gas chromatograph (Agilent Technologies) and a Pegasus III TOF-MS (LECO). The first column was a nonpolar DB5 (30 m × 0.32 mm i.d. × 0.25 *μm* film thickness) from Agilent Technologies, Ltd. (Stockport, U.K.) and the second column a midpolarity BPX50 (4 m × 0.10 mm i.d. × 0.10 *μm* film thickness) from SGE Analytical Science (Milton Keynes, U.K.). The initial temperature of the first dimension column was 60 °C for 2 min, followed by heating at 10 °C *min*^−1^ until 260 °C was reached and held isothermally for a further 3 min. A temperature offset of 20 °C was applied to the second dimension column throughout the GC temperature program. A liquid nitrogen two-stage cold jet modulation system was used, with a modulation period of 5 s and a +15 °C offset from the primary GC oven temperature. Injections were performed with a split ratio of 20:1 and helium was used as a carrier gas with a flow rate of 1 *mLmin*^−1^. The transfer line temperature was 280 °C and the ion source temperature was 250 °C. The analysis was performed using electron ionisation at 70 eV. The spectra were collected at 200 Hz between m/z 45-500, and analyzed using LECO ChromaTOF software.

### Data treatment

PTR-ToF-MS data was analyzed using the Tofware post-processing software (version 2.4.2, TOFWERK AG, Thun, Switzerland; PTR module as distributed by Ionicon Analytik GmbH, Innsbruck, Austria), running in the Igor Pro 6.3 environment (Wavemetrics Inc., Lake Oswego, OR, USA). The results were corrected for fragmentation and converted to mixing ratios as described in Klein *et al*.^23^. Emission factors in *mgkg*^−1^ and concentrations in *μgm*^−3^ were calculated for direct measurements (integrated over the whole experiment) and aging experiments, respectively. The emission factors were corrected for dilution by applying the ratio of CH_4_ before and after dilution. The terpene concentrations were calculated by using the sensitivity of the ions at *m/z* 137 and 205, respectively. By injecting different amounts of *α*-pinene and *β*-caryophyllene in the smogchamber we retrieved the sensitivity of the PTR-ToF-MS for monoterpenes and sesquiterpenes. The different compounds were grouped as described in Klein *et al*.^23^. The new group “terpenes” is the sum of the corrected mass of p-cymene (C_10_H_15_–H^+^, *m/z* 135.117), monoterpenes (C_10_H_16_–H^+^, *m*/*z* 137.132), terpenoids (C_10_H_14_O–H^+^, *m/z* 151.112; C_10_H_16_O–H^+^, *m/z* 153.127) and sesquiterpenes (C_15_H_24_–H^+^, *m/z* 205.195). The HR-ToF-AMS data was treated using Squirrel 1.53G and Pika 1.12G. The organic aerosol mass measured in the smog chamber was wall loss-corrected using the method of Weitkamp *et al*.^37^. To determine the secondary aerosol production potential of the NMOG emissions we calculated the bulk yield (mass of SOA formed divided by initial mass of total NMOG) and an effective yield (mass of SOA formed from the addition of seasoning divided by the mass of terpenes reacted). The contributions of the individual terpene classes to SOA formation were estimated by applying literature yields^24^,^25^ to the measured terpene concentrations and comparing the results with the measured SOA.

## Additional Information

**How to cite this article**: Klein, F. *et al*. Indoor terpene emissions from cooking with herbs and pepper and their secondary organic aerosol production potential. *Sci. Rep*. **6**, 36623; doi: 10.1038/srep36623 (2016).

**Publisher’s note:** Springer Nature remains neutral with regard to jurisdictional claims in published maps and institutional affiliations.

## Supplementary Material

Supplementary Information
