# Supplementary material for: Indoor terpene emissions from cooking with herbs and pepper and their secondary organic aerosol production potential
Source: Sci Rep. 2016 Nov 10;6:36623. doi: 10.1038/srep36623 (PMC5103204; doi:10.1038/srep36623)
Supplement: Supplementary Information [file srep36623-s1.pdf]

Supplementary Material: Indoor terpene emissions from cooking with herbs and pepper and their secondary organic aerosol production potential

Felix Klein[1], Naomi J. Farren[2], Carlo Bozzetti[1], Kaspar R. Daellenbach[1], Dogushan Kilic[1], Nivedita K. Kumar[1], Simone M. Pieber[1], Jay G. Slowik[1], Rosemary N. Tuthill[2], Jacqueline F. Hamilton[2], Urs Baltensperger[1], André S.H. Prévôt[1], and Imad El Haddad[1,*]

[1]Laboratory of Atmospheric Chemistry, Paul Scherrer Institute, Villigen, 5232, Switzerland
[2]Wolfson Atmospheric Chemistry Laboratories, University of York, York, YO10 5DD, UK
[*]imad.el-haddad@psi.ch

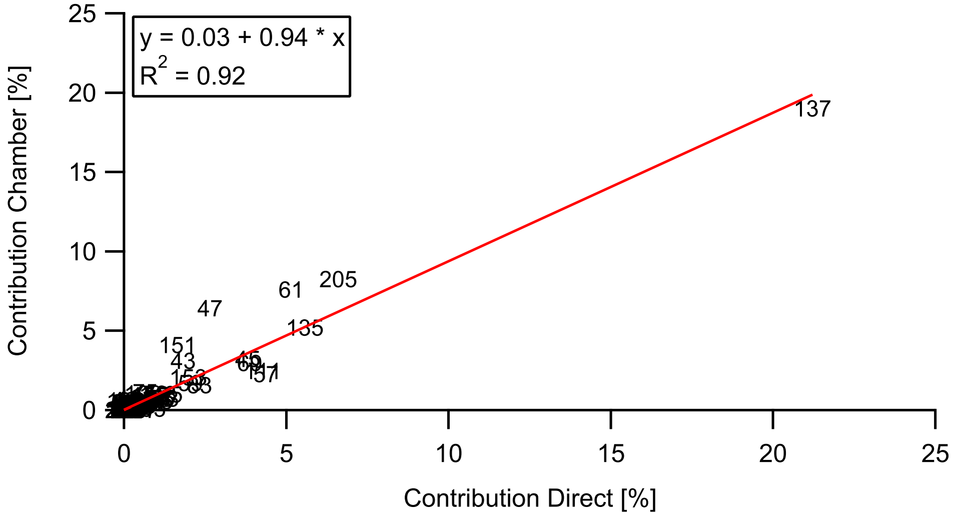

Figure S1. Comparison of PTR-ToF-MS compound contributions from frying meat with herbs and pepper measured directly or from the chamber. Numbers are unit mass *m/z* of the compounds.

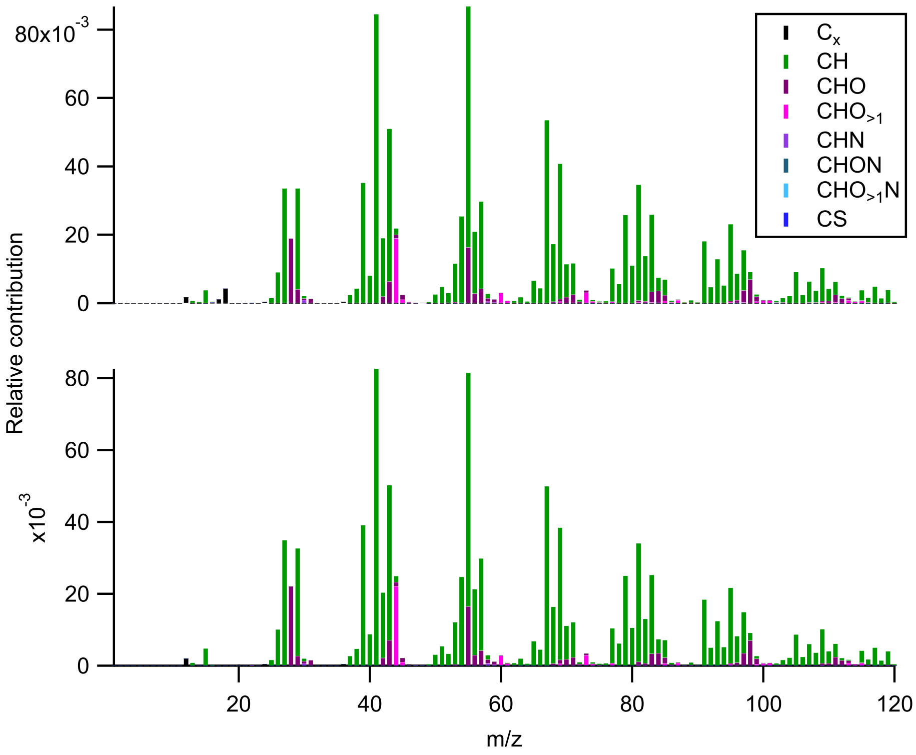

Figure S2. HR-ToF-AMS mass spectra of primary emissions from frying beef with oil alone (upper panel) and from frying beef with herbs and pepper (lower panel).

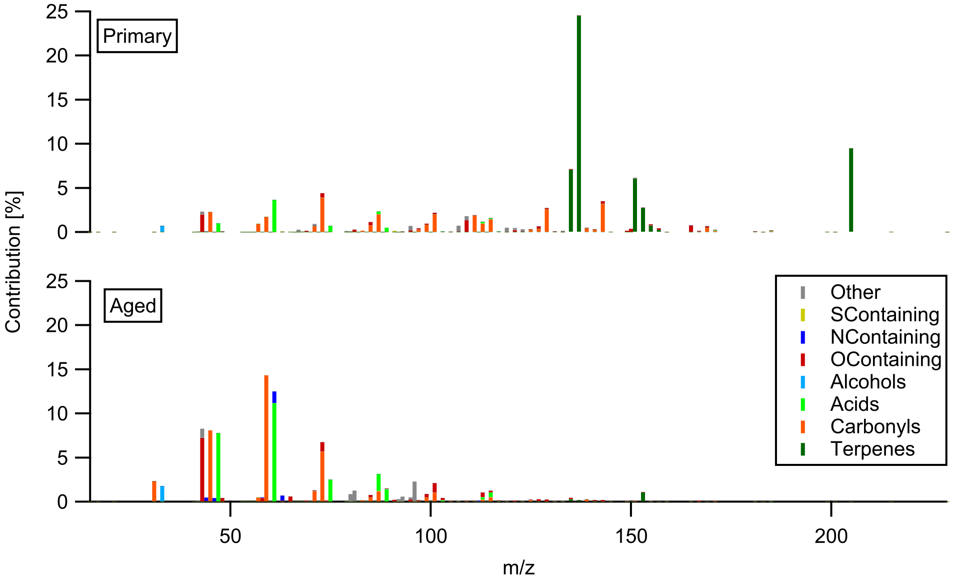

Figure S3. Mass spectra as measured with the PTR-ToF-MS of primary (top) and aged (bottom) emissions from frying meat with herbs and pepper.

Table S1. Compounds identified by GC×GC-ToF-MS measurements for heating herbs to 180°C. RT 1 and RT 2 represent first and second retention time, and similarity is similarity to the reference spectra (out of 1000). *peak coelution prevented similarity match in MS. (MT = Monoterpene; SQT = Sesquiterpene; DIT = Diterpene; OVOC = oxygenated volatile organic compound)

Identified Compound	RT 1 [s]	RT 2 [s]	Similarity	Formula	Type
a-pinene	420	0.82	936	$C_{10}H_{16}$	MT
camphene	435	0.95	887	$C_{10}H_{16}$	MT
sabinene	460	1.01	896	$C_{10}H_{16}$	MT
a-myrcene	470	0.885	902	$C_{10}H_{16}$	MT
a-phellandrene	485	1.01	937	$C_{10}H_{16}$	MT
a-terpinene	495	1.02	894	$C_{10}H_{16}$	MT
p-cymene*	495	1.246	na	$C_{10}H_{16}$	MT
d-limonene	505	1.065	908	$C_{10}H_{16}$	MT
eucalyptol	515	1.135	958	$C_{10}H_{18}O$	OVOC
ocimene	520	0.985	904	$C_{10}H_{16}$	MT
benzylalcohol	520	1.96	931	C_7H_8O	OVOC
g-terpinene	535	1.1	842	$C_{10}H_{16}$	MT
terpinolene	560	1.165	903	$C_{10}H_{16}$	MT
linalool	570	1.16	865	$C_{10}H_{18}O$	OVOC
camphor	620	1.77	916	$C_{10}H_{16}O$	OVOC
Isoborneol	640	1.53	904	$C_{10}H_{18}O$	OVOC
terpinen-4-ol	645	1.435	886	$C_{10}H_{18}O$	OVOC
octanoic acid	650	1.1	848	$C_8H_{16}O_2$	ACID
a-terpineol	655	1.515	933	$C_{10}H_{18}O$	OVOC
estragole	660	1.72	841	$C_{10}H_{12}O$	OVOC
methylsalicylate	660	1.98	869	$C_8H_8O_3$	OVOC
a-santolina alcohol	670	1.44	779	$C_{10}H_{18}O$	OVOC
verbenone	685	1.97	944	$C_{10}H_{14}O$	OVOC
2-furancarboxaldehyde	715	2.59	919	$C_5H_4O_2$	OVOC
m-thymol	725	1.8	891	$C_{10}H_{14}O$	OVOC
thymoquinone	725	1.785	868	$C_{10}H_{12}O_2$	OVOC
dihydroxyacetophenone	735	2.335	850	$C_8H_8O_3$	OVOC
nonanoic acid	740	1.27	868	$C_9H_{18}O_2$	ACID
2-methoxyvinylphenol	765	2.145	947	$C_9H_{10}O_2$	OVOC
a-cubenene	790	1.06	861	$C_{15}H_{24}$	SQT
decanoic acid	795	1.22	849	$C_{10}H_{20}O_2$	ACID
eugenol	800	2.03	896	$C_{10}H_{12}O_2$	OVOC
4-hydroxybenaldehyde	800	2.85	956	$C_7H_6O_2$	OVOC
Unknown	805	2.07	824	$C_{10}H_{12}O$	OVOC
o-thymol	805	1.73	894	$C_{10}H_{14}O$	OVOC
a-copaene	815	1.145	888	$C_{15}H_{24}$	SQT
a-bourbonene	825	1.2	877	$C_{15}H_{24}$	SQT
methyl eugenol	830	2.07	914	$C_{11}H_{14}O_2$	OVOC
niacinamide	835	3.825	953	$C_6H_6N_2O$	AMIDE
b-caryophyllene	850	1.34	900	$C_{15}H_{24}$	SQT
trans-bergamotene	855	1.14	916	$C_{15}H_{24}$	SQT
Z-E-farsenene	855	1.32	783	$C_{15}H_{24}$	SQT
Unidentified	870	2.125	na	$C_{10}H_{14}O_2$	OVOC
humulene	875	1.41	907	$C_{15}H_{24}$	SQT
a-amorphene	890	1.358	887	$C_{15}H_{24}$	SQT
a-longipinene	890	1.2	810	$C_{15}H_{24}$	SQT
a-muurolene	905	1.395	878	$C_{15}H_{24}$	SQT
a-bisabolene	910	1.215	895	$C_{15}H_{24}$	SQT

Table S1. Continued: Compounds identified by GC×GC-ToF-MS measurements for heating herbs to 180°C. RT 1 and RT 2 represent first and second retention time, and similarity is similarity to the reference spectra(out of 1000). *peak coelultion prevented similarity match in MS.

Identified Compound	RT 1 [s]	RT 2 [s]	Similarity	Formula	Type
cadinene	925	1.405	880	$C_{15}H_{24}$	SQT
caryophyllene alcohol	965	1.695	821	$C_{15}H_{26}O$	OVOC
caryophyllene oxide	975	1.795	879	$C_{15}H_{24}O$	OVOC
cadinol	1010	1.71	834	$C_{15}H_{26}O$	OVOC
methyl-jasmonate	1010	2.16	922	$C_{13}H_{20}O_3$	OVOC
a-bisabolol	1035	1.575	875	$C_{15}H_{26}O$	OVOC
tetradecanoic acid	1075	1.31	882	$C_{14}H_{28}O_2$	ACID
pentadecanoic acid	1135	1.32	848	$C_{15}H_{30}O_2$	ACID
rimuene	1190	1.875	711	$C_{20}H_{32}$	DIT
hexadecanoic acid	1200	2.36	853	$C_{16}H_{32}O_2$	ACID
cembrene	1270	3.87	777	$C_{20}H_{32}$	DIT
Phytol	1350	2.415	900	$C_{20}H_{40}O$	OVOC
linolenic acid	1365	3.57	894	$C_{18}H_{30}O_2$	ACID
octadecanoic acid	1380	3.05	850	$C_{18}H_{36}O_2$	ACID

Table S2. Compounds identified by GC×GC-ToF-MS measurements for heating pepper to 180°C. RT 1 and RT 2 represent first and second retention time, and similarity is similarity to the reference spectra (out of 1000). (MT = Monoterpene; SQT = Sesquiterpene; DIT = Diterpene; OVOC = oxygenated volatile organic compound; HC = Hydrocarbon)

Identified Compound	RT 1 [s]	RT 2 [s]	Similarity	Formula	Type
a-thujene	410	0.8	867	$C_{10}H_{16}$	MT
a-pinene	425	0.82	948	$C_{10}H_{16}$	MT
camphene	435	0.935	951	$C_{10}H_{16}$	MT
sabinene	455	0.96	912	$C_{10}H_{16}$	MT
carene	480	0.98	910	$C_{10}H_{16}$	MT
a-phellandrene	485	0.985	793	$C_{10}H_{16}$	MT
d-limonene	505	1.115	862	$C_{10}H_{16}$	MT
cis-a-ocimene	520	0.995	922	$C_{10}H_{16}$	MT
g-terpinene	530	1.15	886	$C_{10}H_{16}$	MT
dimethylstyrene	560	1.475	945	$C_{10}H_{12}$	HC
linalool	565	1.21	890	$C_{10}H_{18}O$	OVOC
1,3,8-p-methatriene	580	1.375	919	$C_{10}H_{14}$	HC
unknown	605	1.45	na	$C_{10}H_{14}$	HC
camphor	615	1.795	942	$C_{10}H_{16}O$	OVOC
octanoic acid	625	1.25	617	$C_8H_{16}O_2$	ACID
geranaldehyde	635	1.575	746	$C_{10}H_{18}O$	OVOC
terpinen-4-ol	645	1.44	879	$C_{10}H_{18}O$	OVOC
3-pinanone	645	1.765	822	$C_{10}H_{16}O$	OVOC
p-cymen-8-ol	650	1.775	897	$C_{10}H_{14}O$	OVOC
4(10)-thujen-3-ol acetate	665	1.68	878	$C_{12}H_{18}O_2$	OVOC
cis-geraniol	675	1.515	791	$C_{10}H_{18}O$	OVOC
E-3(10)-caren-4-ol	675	1.675	838	$C_{10}H_{16}O$	OVOC
p-cuminic aldehyde	700	1.92	829	$C_{10}H_{12}O$	OVOC
p-menth-1-en-3-one	710	1.915	919	$C_{10}H_{16}O$	OVOC
eucarvone	710	2.06	852	$C_{10}H_{14}O$	OVOC
nonanoic acid	710	1.23	827	$C_9H_{18}O_2$	ACID
isoestragole	735	1.91	804	$C_{10}H_{12}O$	OVOC
2-undecanone	735	1.19	893	$C_{11}H_{22}O$	OVOC

Table S2. Continued: Compounds identified by GC×GC-ToF-MS measurements for heating pepper to 180°C. RT 1 and RT 2 represent first and second retention time, and similarity is similarity to the reference spectra(out of 1000).

Identified Compound	RT 1 [s]	RT 2 [s]	Similarity	Formula	Type
Thymol	745	1.82	859	$C_{10}H_{14}O$	OVOC
cis-verbenone	760	2.39	883	$C_{10}H_{14}O$	OVOC
piperonal	775	3.055	956	$C_8H_6O_3$	OVOC
elemene	780	1.05	755	$C_{15}H_{24}$	SQT
a-cubenene	790	1.045	868	$C_{15}H_{24}$	SQT
decanoic acid	790	1.245	843	$C_{10}H_{20}O_2$	ACID
copaene	805	1.2	890	$C_{15}H_{24}$	SQT
Unknown SQT	815	1.17	na	$C_{15}H_{24}$	SQT
2,3-epoxycarane	820	2.185	772	$C_{10}H_{16}O$	OVOC
a-gurjunene	840	1.25	873	$C_{15}H_{24}$	SQT
b-caryophyllene	855	1.36	908	$C_{15}H_{24}$	SQT
a-farsenene	865	1.13	928	$C_{15}H_{24}$	SQT
humulene	875	1.495	720	$C_{15}H_{24}$	SQT
a-bisabolene	905	1.32	902	$C_{15}H_{24}$	SQT
Unknown SQT	905	1.505	na	$C_{15}H_{24}$	SQT
cadinene	920	1.515	878	$C_{15}H_{24}$	SQT
undecanoic acid	935	1.385	871	$C_{11}H_{22}O_2$	ACID
caryophyllene oxide	965	1.855	781	$C_{15}H_{24}O$	OVOC
cubenol	985	1.83	798	$C_{15}H_{26}O$	OVOC
Unknown SQT	995	2.46	833	$C_{15}H_{24}$	SQT
lanceol	995	2.61	801	$C_{15}H_{24}O$	OVOC
farsenene epoxide	1000	3.045	760	$C_{15}H_{24}O$	OVOC
unknown	1000	3.12	na	$C_{15}H_{22}$	HC
unknown	1030	3.31	na	$C_{13}H_{16}O_2$	OVOC
trans-2-a-bisabolene epoxide	1045	3.82	833	$C_{15}H_{24}O$	OVOC
unknown oVOC	1050	3.495	na	$C_{15}H_{26}O$	OVOC
hexadecanoic acid	1280	3.12	846	$C_{16}H_{32}O_2$	ACID

Table S3. Amount of seasoning added for the different experiments

	Pepper [g]	Herbs [g]
Meat	0	0
Pepper	2	0
Herbs	0	4
Both low	0.5	1
Both mid	1	2
Both high	2	4

Table S4. Emission factors [$\mu g/m^{-3}$] from frying processes using different amounts of seasoning (g Herbs/Pepper). S-Containing and N-Containing compounds are always below detection limit.

	Amount	Terpenes	Carbonyls	Acids	Alcohols	O-Containing	Other	Total NMOG
Meat	0/0	0.5 +- 0.5	10.5 +- 7.5	0.5 +- 0.3	0	1.7 +- 1	0.8 +- 0.4	14.1 +- 9.6
Both low	1/0.5	2.6 +- 1.3	10.4 +- 0.7	0.3 +- 0	0.2 +-0.2	1.5 +- 0.1	1 +- 0.3	16 +- 2.5
Both mid	2/1	6.8 +- 2.5	11.9 +- 3.4	0.5 +- 0	0.3 +- 0.1	1.7 +- 0.6	1.3 +- 0.4	22.5 +- 7.0
Both high	4/2	12.3 +- 0.6	10.3 +- 4.3	0.6 +- 0	0.3 +- 0.1	2.9 +- 0.4	2 +- 0.1	28.4 +- 4.1
Herbs	4/0	9 +- 2.6	14.7 +- 12.8	0.7 +- 0.5	0.5 +- 0.2	2.7 +- 1.8	1.8 +- 0.8	29.4 +- 18.6
Pepper	0/2	4.4 +- 1	9.5 +- 4.1	0.7 +- 0.3	0	1.6 +- 0.8	0.9 +- 0.3	17.2 +- 3.9
